# Effect of vertical vibration stimulation at different frequencies on delayed muscle soreness in athletes: A randomized trial

**DOI:** 10.3389/fpubh.2022.980454

**Published:** 2022-10-14

**Authors:** Liang Cheng, Kun Wang, Benxiang He, Yang Yan

**Affiliations:** ^1^School of Sports Medicine and Health, Chengdu Sport University, Chengdu, China; ^2^Human Movement Science, Sichuan Sports College, Chengdu, China

**Keywords:** athletes, delayed muscle soreness, vibration, pain index, peak torque, serum index

## Abstract

**Background:**

The effect of stimulation with different vibration frequencies on delayed muscle soreness (DOMS) has not yet been determined. This study was conducted to investigate the effect of medium- and high-frequency vertical vibration stimulation on DOMS in track and field athletes.

**Methods:**

A total of 38 elite men's track and field athletes were recruited during the off-season. Through the digital randomization method, the participants were divided into three groups. Two-knee DOMS modeling was performed on the medium-frequency group (MFG, 25 Hz, *n* = 13), high-frequency group (HFG, 50 Hz, *n* = 12) and control group (CG, 0 Hz, *n* = 13). The three groups were matched in age, height and body mass. Vertical vibration stimulation was performed for 10 min on the MFG and HFG. Visual analog score (VAS); interleukin-6 (IL-6), lactic dehydrogenase (LDH) and creatine kinase (CK) levels and knee extension peak torque (PT) were determined immediately and at 24, 48, and 72 h after vibration.

**Results:**

The 48 and 72 h VASs of the HFG were lower than those of the MFG and CG. The immediate and 24 and 48 h IL-6 concentrations in the HFG were lower than those in the CG. The 24 h IL-6 concentration in the HFG was lower than that in the MFG and that in the MFG was lower than that in the CG. LDH concentrations at different time points did not differ amongst groups. Immediate and 24 h CK concentrations were lower in the HFG than in the CG. The immediate and 24, 48, and 72 h knee extensions at 60°/s PT were lower in the HFG than in the CG. The immediate and 24 and 48 h knee extension at 60 °/s PT in the MFG were lower than those in the CG. The 24 h knee extension (240°/s peak torque) value in the HFG was lower than that in the CG.

**Conclusion:**

Stimulation with 50 Hz (amplitude of 3 mm) vibration can reduce the muscle pain, IL-6 and CK concentrations and knee extension loss caused by DOMS. However, stimulation with 25 Hz (amplitude of 3 mm) vibration had poor effects. Results suggested that high-frequency vibration training is an effective strategy for relieving DOMS after intensive training.

## Introduction

Delayed onset muscle soreness (DOMS) easily occurs after the performance of unaccustomed sports or unaccustomed movements. DOMS occurs within 8–24 h after intense centrifugal movement, it peaks within 72 h, gradually eases and recovers within approximately 1 week (1, 2). Several hypotheses for the mechanisms of DOMS development exist and are based on lactic acid accumulation, muscle spasms, connective tissue and muscle injuries, inflammation and enzyme loss (3, 4). The elimination or relief of DOMS mainly involves drugs (anti-inflammatory drugs or antioxidants), general physical therapy (adequate warm-up and relaxation, hot and cold therapy, electric therapy, electrical stimulation and ultrasound or electric ion input) and Chinese medicine methods (acupuncture, massage or Chinese medicine) (5–7). However, a highly effective method for DOMS relief has not been found.

A meta-analysis of 10 randomized controlled trials involving 258 ordinary young subjects showed that vertical vibration stimulation improved the visual analog scale (VAS) at 24, 48, and 72 h and reduced creatine kinase (CK) concentrations at 24 and 48 h (8). In ordinary young people subjected to immediate DOMS modeling of the elbow, vertical vibration stimulation at 50 Hz for 5 min (9), 20 Hz for 2 min (10) and 20 Hz for 30 min (11) reduced serum lactate dehydrogenase (LDH) and CK concentrations at 48 h compared with the CG (9). The maximum contraction force was increased at 72 h (10).

The increase in the 48 and 96 h joint ranges reduced the immediate pain index (11). In ordinary male college students who underwent DOMS modeling of the knee joint, immediate vertical vibration stimulation (20–45 Hz for 10 min) reduced the immediate and 24 and 48 h pain index scores (12). In these studies (8–12), the DOMS model was constructed in the general population and treated with vibration within the frequency range of 20–50 Hz. The indicators of the intervention were the pain index, joint activity, maximum contractions and serum LDH and CK levels immediately and at 24, 48, and 72 h after vibration.

Reports on DOMS in athlete groups and baseline data for experimental designs are lacking, and the effect of stimulation with different vibration frequencies on DOMS has not yet been determined. Given this situation, current study attempted to explore the effects of different vibration frequencies of the same amplitude on DOMS to provide a basis for reducing DOMS and thus decrease the risk of injury in athletes. This study assumed that the two vibration frequencies of 20 and 50 Hz relieved DOMS symptoms in athletes.

## Materials and methods

### Participants

The present study comprised a 3 (number of groups) × 5 (number of measurements) experimental design. Referring to the results of previous studies (13), Using G-power software with the effffect size set at 0.3, power set at 0.8, and α set at 0.05, the calculated required sample size was at least 36 subjects. A total of 38 elite men's track and field athletes was recruited during the off-season. Participants were included if they were aged 18–25 years old. This study complied with the Helsinki Declaration, and the participants provided signed informed consent. The patients were excluded if they had lower limb joint injury and had undergone systematic exercise training in the last 1 week. Through the digital randomization method, the participants were divided into the medium-frequency group (MFG, *n* = 13), high-frequency group (HFG, *n* = 12) and control group (CG, *n* = 13). No significant differences in age, height and body mass were found amongst the participants (Table 1). This study was approved by the Sichuan Sports College ethics committee (No: 202101).

**Table 1:** Basic information of the study participants.

**Group**	**Medium frequency group** **(*n* = 13)**	**High frequency group** **(*n* = 12)**	**Control group** **(*n* = 13)**
Age (years)	18.8 ± 3.2	18.5 ± 2.9	19.3 ± 4.2
Height (cm)	178.3 ± 5.8	178.1 ± 8.2	177.8 ± 6.6
Body mass (kg)	68.9 ± 6.0	69.1 ± 8.2	68.7 ± 7.9
Years of training (years)	7.8 ± 2.7	7.7 ± 3.9	8.1 ± 3.5

### Knee DOMS modeling

The experimental design for the DOMS modeling of the knee joint was used as a reference (13). Firstly, the maximum oxygen intake test was conducted on all subjects (Metalyzer 3B cardiopulmonary function test system; Cortex Medical, Leipzig, Germany). Detailed records were taken. After 1 week of DOMS modeling, the subject was instructed to run downhill on a treadmill (Model NETL 28717; ICON, San Francisco, CA, USA) at a slope of −10° in five groups for 8 min/group with a 2 min flat walk between groups. Exercise intensity matching for each subject was based on the maximum oxygen intake to ensure that the included subjects with DOMS modeling had similar aerobic capacities and maintained similar strengths during downhill runs. The subjects wore Polar tables (Finland, model: M430) to monitor that their heart rate remained at 80% during downhill runs. The treadmill was adjusted if it was too low or too high. The subjects were followed up within 72 h of DOMS modeling to ensure that they had stopped performing other training activities or treatment. The entire DOMS modeling process lasted for 7 days, and given the long duration of DOMS modeling, approximately 5–6 cases were completed per day.

### Vibratory stimulation intervention

The subjects in the MFG were treated at a frequency of 25 Hz and amplitude of 3 mm and those in the HFG were treated at a frequency of 50 Hz and amplitude of 3 mm. An American Power-Plate vibrating platform (Power Plate^®^ pro5™) was used to complete 10 min of DOMS stimulation (11, 14–17) on the vibration group (the MFG and the HFG). Under vertical vibration stimulation at different frequencies, the subject did half squats and lunge pulls on the vibratory platform (Perform the half squats movement of both legs for 30 s, and lunge pulls the left and right legs for 60 s, and cycle six groups). The vibrator was turned off for the control group, who completed the same actions for the same number of repetitions and durations as the vibration group (18). The three groups underwent strength tests, and their VASs; IL-6, LDH and CK concentrations and knee extensions at different angular velocities (60°/s and 240°/s) were determined before vibration stimulation (baseline) and immediately and 24, 48, and 72 h.

### VAS test

The VAS test was performed to assess the subjective pain in the quadriceps of all subjects. A 10 cm line was printed on white paper. The subjects were informed that 0 cm represented ‘no pain’ and 10 cm represented ‘most painful’. In accordance with their subjective feelings, the subject drew a vertical line on their left and right femoral quadriceps. The corresponding length was the pain score (0–10 points). This measurement method has been validated in our previous study (18, 19). For discussion, this study averaged the left and right VASs.

### IL-6, LDH, and CK tests

Approximately 4 ml of venous blood was collected with a RT−9600 automatic biochemical analyser to determine the LDH and CK concentrations at five time points. Kits were provided by Shanghai Lanxing Biotechnology Co., Ltd. Serum IL-6 was tested via a double antibody sandwich enzyme-linked immunoassay with kits provided by Shanghai Varan Biotechnology Co., Ltd.

### Knee extension equal muscle strength test

All of the subjects' knee joints (with 10–90° range of motion) were tested three times each at 60°/s and 240°/s by using an IsoMed 2000 muscle tester. The torso and hip of the subject in a seated position and in knee extension mode were fixed with wide bands; the peak torque (PT) measurement reflect joints muscle strength at different angular speeds (19–21). Participants were verbally encouraged throughout the test process to ensure reliable test data.

### Data analysis

The test data of the three groups at five time points (baseline; immediately after vibration and at 24, 48, and 72 h after vibration) were presented as mean ± standard deviation by using SPSS 20.0. Two-factor variance analysis with a mixed design was used to test whether the group, the main effect of time and the group (3) and time (5) interacted. Differences between groups were compared via single-factor variance analysis if the group and time interacted. If time had the main effect, different time points were compared. If the group had no main effect, the differences between the groups were compared. Bonferroni adjustment was performed to ensure that the overall I type rate per variance analysis did not exceed 0.05. The significance level was *P* = 0.05.

## Results

The two-factor variance analysis of the hybrid design revealed that IL-6 and CK concentration interacted with time in the 60°/s PT group, and the single-factor variance analysis group showed repeated differences (*P* < 0.01) in VAS, LDH concentration and knee extension at 240°/s. Further analysis showed that group and time had the main effect. Bonferroni analysis was performed to compare different time points and the same time points between groups. The intergroup comparison of baseline data revealed no statistically significant differences for VAS (*F*_(2, 35)_ = 0.277, *P* = 0.760), IL-6 concentration (*F*_(2, 35)_ = 0.131, *P* = 0.878), LDH concentration (*F*_(2, 35)_ = 0.041, *P* = 0.959), CK concentration (*F*_(2, 35)_ = 0.235, *P* = 0.791), knee extension at 60°/s PT (*F*_(2, 35)_ = 0.254, *P* = 0.717) and knee extension at 240 °/s PT (*F*_(2, 35)_ = 0.089, *P* = 0.915).

### VAS

**Figure 1:**
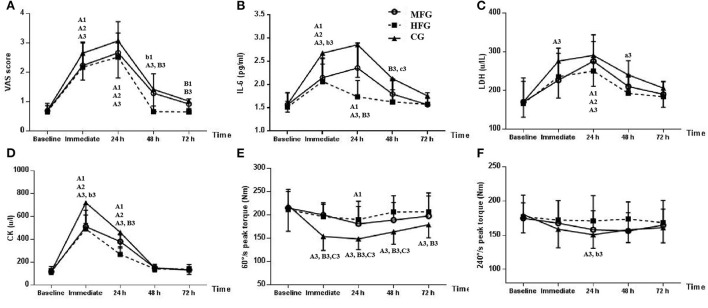
Changs in the three groups at different time points. MFG, medium-frequency group; HFG, high-frequency group; CG, control group. Values at different time points were compared with the baseline in the same group: a1 (MFG), a2 (HFG), and a3 (CG) indicate *P* < 0.05; A1 (MFG), A2 (HFG), and A3 (CG) indicate *P* < 0.01. MFG or CG was compared with HFG at the same point in time: b1 (MFG) and b3 (CG) indicate *P* < 0.05. B1 (MFG) and B3 (CG) indicate *P* < 0.01. CG was compared with MFG at the same point in time. c3 (CG) indicates *P* < 0.05. **(A)** VAS score. **(B)** IL-6. **(C)** LDH. **(D)** CK. **(E)** 60 °/s peak torque. **(F)** 240 °/s peak torque.

Figure 1A shows that the VAS of the three groups gradually decreased at 24 h after vibration. Compared with the baseline VAS, the immediate (95% CI: 0.8170–2.2600) and 24 h (95% CI: 1.2477–2.6907) (*P* < 0.001) VASs of the MFG increased; the immediate (95% CI: 0.9948–2.0052) and 24 h (95% CI: 1.3448–2.3552) (*P* < 0.001) VASs of the HFG increased and the immediate (95% CI: 1.3383–2.5541), 24 h (95% CI: 1.7690–2.9848), and 48 h (95% CI: 0.1229–1.3387) (*P* < 0.001) VASs of the CG increased. Intergroup comparisons showed no statistical difference between the immediate and 24 h VASs. The 48 h VAS of the HFG was lower than that of the MFG (*P* = 0.018, 95% CI: −1.1616–−0.0896) and CG (*P* = 0.003, 95% CI: −1.3001–−0.2281). The 72 h VAS of the HFG was significantly lower than that of the MFG (*P* = 0.006, 950% CI: −0.4351–−0.0610) and CG (*P* < 0.001, 95% CI: −0.5351–−0.1610).

### IL-6 concentration

Figure 1B shows that the IL-6 concentration in the MFG and CG peaked after DOMS modeling, whereas that in the HFG peaked and then decreased immediately. The immediate (95% CI: 0.1576–1.0270) and 24 h (95% CI: 0.3576–1.2270) (*P* < 0.01) IL-6 concentration in the MFG; the immediate (95% CI: 0.1898–0.9436) (*P* < 0.001) IL-6 concentration in the HFG and the immediate (95% CI: 0.5046–1.6954) and 24 h (95% CI: 0.6969–1.8877) (*P* < 0.001) IL-6 concentration in the CG had all increased relative to the baseline IL-6 concentration. Intergroup comparison revealed that the immediate (*P* = 0.039, 95% CI: −1.1628–−0.0244), 24 h (*P* < 0.001, 95% CI: −1.6356–−0.6029), 48 h (*P* = 0.004, 95% CI: −0.8541–−0.1408) IL-6 concentrations in the HFG were less than those in the CG. The 24 h (*P* = 0.016, 95% CI: −1.1279– −0.0952) IL-6 concentration in the HFG was less than that in the MFG (*P* = 0.049, 95% CI: 0.0018–1.0136) and was statistically lower than that in the CG. The 72 h IL-6 concentration did not statistically differ amongst groups.

### LDH concentration

Figure 1C illustrates that the LDH concentrations in the MFG and CG peaked after DOMS modeling, whereas those in the HFG decreased at 24 h. The 24 h (95% CI: 34.4721–176.6048) (*P* = 0.001) LDH concentration in the MFG; the immediate (95% CI: 28.8319–108.0015) and 24 h (95% CI: 43.7485–122.9181) (*P* < 0.001) LDH concentrations in the HFG and the immediate (*P* = 0.001, 95% CI: 34.5385–174.0769), 24 h (*P* < 0.001, 95% CI: 51.1538–190.6923) and 48 h (*P* = 0.049, 95% CI: 0.1538–139.6923) LDH concentrations in the CG had increased compared with the baseline LDH concentration. Intergroup comparisons showed no statistical difference between groups at different points.

### CK concentration

Figure 1D depicts that the CK concentrations in the MFG, HFG and CG peaked then decreased immediately after DOMS modeling. The immediate (95% CI: 225.3390–562.5072) and 24 h (95% CI: 94.9544–432.1225) (*P* < 0.001) CK concentrations in the MFG; the immediate (95% CI: 256.5230–470.3103) and 24 h (95% CI: 35.7730–249.5603) (*P* < 0.001) CK concentrations in the HFG and the immediate (95% CI: 465.1134–734.8866) and 24 h (95% CI: 204.9595–474.7328) (*P* < 0.001) CK concentrations in the CG had increased compared with the baseline CK concentration. Intergroup comparison indicated that the immediate (*P* = 0.040, 95% CI: 8.1910–454.0911) and 24 h (*P* = 0.005, 95% CI: 51.2472–332.2271) CK concentrations in the HFG were statistically significantly lower than those in the CG. No statistical differences in the 48 and 72 h CK concentrations were found between groups.

### Knee extension at 60°/s PT

Figure 1E shows that the PT of the MFG, HFG and CG decreased after DOMS modeling. The immediate (95% CI: 43.1258–88.2588), 24 h (95% CI: 49.1258–94.2588), 48 h (95% CI: 33.4335–78.5665) and 72 h (95% CI: 15.7412 – 60.8742) (*P* < 0.001) knee extensions at 60°/s PT in the CG and the 24 h (*P* = 0.006, 95% CI: 6.4327–60.1827) knee extension at 60°/s PT of the HFG decreased had decreased compared with the baseline knee extension at 60°/s PT. Intergroup comparisons demonstrated that immediate (*P* < 0.001, 95% CI: 20.0715–63.9926), 24 h (*P* < 0.001, 95% CI: 24.7012–59.0296), 48 h (*P* < 0.001, 95% CI: 22.4126–62.9336) and 72 h (*P* = 0.024, 95% CI: 2.6707–46.6241) knee extensions at 60°/s PT in the HFG were significantly higher than those in the CG. Immediate (*P* < 0.001, 95% CI: 23.0985–66.1323), 24 h (*P* < 0.001, 95% CI: 16.4903–50.1251) and 48 h (*P* = 0.007, 95% CI: 5.7926–44.5151) knee extensions at 60°/s PT in the MFG were significantly greater than those in the CG.

### Knee extension at 240°/s PT

Figure 1F shows that knee extensions at 240°/s PT in the MFG, HFG and CG decreased after DOMS modeling. Compared with the baseline knee extension at 240°/s PT, the knee extensions at 240°/s PT at each time point in the MFG and HFG were not significantly different, whereas the 24 h knee extension at 240°/s PT in the CG had decreased (*P* = 0.002, 95% CI: 7.8258–52.9588).

Intergroup comparison revealed that the 24 h (*P* = 0.014, 95% CI: 3.4012–37.7219) 240°/s PT in the HFG were significantly higher than those in the CG. No statistical difference was found in the 48 and 72 h knee extensions at 240°/s PT amongst the three groups.

## Discussion

This study attempted to explore the effect of stimulation with vibration at different frequencies and the same amplitude on DOMS in athletes to provide a theoretical basis for reducing post-training DOMS in athletes with vertical vibration stimulation.

### VAS

This study used VAS to test the pain response in the femoral quadriceps at different periods subjectively and found a significant reduction in the 48 and 72 h VASs of the HFG compared with those of the CG. The VASs of the MFG at different time points after DOMS modeling were lower than those in the CG without statistical significance. In addition, the VASs of the three groups decreased first at 24 h after vibration. The 48 h VASs of the HFG approached the baseline. This result indicated that the HFG recovered from pain faster than the other groups.

Inflammatory reactions can increase pain sensation, whereas vibratory stimulation can reduce muscle pain. A series of studies on subjects who underwent DOMS modeling found that immediate vibrational stimulation exerted positive effects. Wheeler et al. (12). found that stimulation at 20–45 Hz for 10 min resulted in a significant reduction in the immediate and 24 and 48 h VASs of ordinary male college students subjected to DOMS knee joint modeling. Stimulation with the vibration frequency of 20 Hz for 30 min significantly reduced the immediate VASs of ordinary male college students who underwent DOMS elbow modeling (13). Vertical vibration stimulation at the frequency of 30 Hz for 10 min resulted in a significant decrease in the 96 and 120 h VASs of ordinary male subjects who were subjected to the DOMS modeling of their buttocks and thighs (14). Treatment with vibration at the frequency of 5 Hz for 15 min reduced the immediate VASs of subjects with DOMS of the lower limbs [16]. Another study reported that multiple vertical vibration stimulations at different times (2 times/day vibratory stimulation at 30 Hz for 10 min within 3 days after exercise) resulted in a significant decrease in the 12, 24, 48, and 72 h VASs of middle-aged men who were subjected to the DOMS modeling of the lower limbs (15).

In contrast to previous studies (11–16), the current study compared two vibrations at different frequencies and found that vertical vibration stimulation at 50 Hz decreased 48 and 72 h muscle pain, whereas that at 25 Hz did not result in statistically significant changes. These different results may be related to the use of different modeling objects. Previous studies involved ordinary people, whereas this study involved elite athletes who may be more adaptable to vertical vibration stimulation than ordinary people.

Vertical vibration stimulation may reduce pain in DOMS via several mechanisms. Firstly, in the human body, vibration treatment stimulates muscle shuttling and α-motor neurons and then induces strong muscle contraction and pain perception response to reduce pain perception (22). Secondly, vertical vibration stimulation can relieve pain by activating large-diameter fibers and inhibiting small-diameter fibers (23). Thirdly, in subjects with DOMS, vibration treatment induces proprioceptor feedback for pain inhibition, accelerates peripheral nerve vibration receivers in skin tissue, stimulates inhibitory intermediate neurons in spinal cord nerves, reduces the transmission of pain perception messages by A-δ and C nerve cells from the spinal cord to the brain and then reduces muscle pain (24).

### IL−6 concentration

This study tested the changes in IL-6 concentrations at different time points to measure the muscle injury caused by DOMS in athletes. High IL-6 concentrations are indicative of strong acute inflammatory response and considerable muscle injury. This study showed that the immediate and 24 and 48 h IL-6 concentrations in the HFG decreased significantly compared with those in the CG, and only the 24 h IL-6 concentration in the MFG decreased. These results demonstrated that high-frequency vertical vibration stimulation reduced the acute inflammatory response caused by DOMS and accelerated body recovery. By contrast, the medium-frequency vertical vibration stimulation had a poor effect.

IL-6 is a cell target that is mainly produced by a variety of cells, such as monuclemacrophages. It participates in a series of complex biological reactions in the human body and often plays a role in inflammation, immune response and bone metabolism. At the same time, it is also a strong pain-causing inflammatory response factor that can serve as one of the important indicators of inflammatory response (25). Previous works have found that vibratory stimulation at 30 Hz for 10 min reduced 24 and 120 h IL-6 concentrations, thus reducing the inflammatory response caused by DOMS (14). This study explored the effects of vibrational stimuli at different frequencies on IL-6 and expands previous study (14). It found that medium- and high-frequency stimulation can cause significant changes in IL-6 concentrations at 24 and 48 h after DOMS modeling, respectively.

Mechanistic explorations have found that inflammation and inflammatory reactions can cause pain perception, and IL-6 can inhibit anti-inflammatory cytokines and reduce neurological pain (26). Vibration stimulation reduces pain by increasing the discharge of human muscles, wherein oxygen consumption is proportional to vibration intensity; increasing local muscle temperature and skin blood flow; promoting blood and lymphatic circulation; accelerating the inflammatory reaction and suppressing the release of IL-6 inflammatory factors (26). Furthermore, in this study, the IL-6 concentrations in the three groups first increased and then decreased, with those in the MFG peaking at 24 h and approaching baseline levels at 72 h and those in the HFG peaking immediately and approaching baseline levels at 48 h, indicating that high-frequency vibratory stimulation is highly effective for inflammatory recovery.

### CK and LDH concentrations

CK is primarily found in human skeletal muscle and myocardial cells, catalyzing the breakdown of phosphoric creatine and providing energy for muscle contraction. LDH is an important indicator of muscle injury (27). Muscle injury is mainly related to exercise intensity (28). In this study, CK and LDH mainly originated from skeletal muscle given that DOMS modeling was performed on double knee extension in athletes. When the subject experienced DOMS and muscle tissue damage, the permeability of the skeletal muscle cell membrane changed, and CK and LDH increased in the blood. We suggest that highly pronounced DOMS symptoms are associated with high CK and LDH concentrations.

This study found that immediate CK concentration in the HFG decreased significantly relative to that in the CG. However, CK concentration did not show statistically significant changes at 48 and 72 h after vibration. Furthermore, the immediate and 24 and 48 h LDH concentrations of the MFG, HG and CG did not significantly differ. The CK and LDH concentrations of the three groups increased first before decreasing. The CK of the MFG and HFG was close to the baseline level at 48 h. However, the CG did not approach the baseline level until 72 h. peaked at immediately (LDH) and 24 h (CK) after vibration. The LDH of the three groups did not return to baseline levels after 72 h. However, the immediate LDH levels of the MFG and FMG were less than those of the CG. Medium- and high-frequency vertical vibration stimulation can decrease the increase in CK and LDH caused by DOMS. One study suggested that vibrational stimulation at 50 Hz for 5 min significantly reduced the immediate and 48 h CK and LDH concentrations in ordinary young women (9). In the DOMS model of average male/female thighs, the immediate and 24 and 48 h CK showed a significant decrease after vertical vibration stimulation at 35 Hz for 12 min (17). In common male/female quadriceps subjected to DOMS modeling, vibrational stimulation at 50 Hz for 30 min resulted in a significant decrease in the immediate and 12 and 24 h CK (29). Vibrational stimulation can promote local blood and lymphatic circulation in subjects; cause CK and LDH to enter rapidly into the body circulation with the lymphatic circulation; accelerate the recovery from DOMS inflammatory response and promote the repair and remodeling of damaged muscle tissue (29).

### Knee joint at 60 and 240°/s PT

In contrast to previous works, this study utilized an isovelocity muscle force test system to analyse the variations in different angular velocities at the DOMS sites of athletes (10, 27). Muscle strength was measured differently. Previous studies analyzed the variation in the maximum isolength contraction force on the DOMS site in ordinary youths and showed that vertical vibration stimulation at 50 Hz for 5 min significantly increased the 72 h maximum contraction force on the elbows of average young women (6). Another study suggested that vibratory stimulation at 50 Hz for 30 min significantly increased the 24 h maximum systolic retraction in the average male/female femoral quadriceps (29). This study demonstrated that the PT of 60°/s and 240°/s were reduced in the three groups after DOMS modeling, and medium- and high-frequency vertical vibration stimulation could significantly increase the 48 h PT relative to the immediate PT. Notably, only high-frequency stimulation could significantly reduce the loss in knee extension muscle at 240°/s PT at 24 h after vibration through a mechanism that may be related the increase in motor unit activation and muscle tension cause by vertical vibration stimulation (30, 31). This study suggested that vibratory stimulation improves blood and lymphatic circulation at the DOMS site, accelerates recovery from inflammatory reactions, reduces muscle pain, induces athletes to mobilize additional muscle fibers during isometric muscle force and reduces the loss of muscle strength caused by DOMS (4, 32).

This study verified its hypothesis but has certain limitations. First, because the research object is elite athletes, we can't add more samples, which may limit the universality of the conclusion; Secondly, we only used the amplitude of 3 mm, and did not consider the impact of different amplitudes on the results. Further research is needed in the future.

## Conclusion

Stimulation with 50 Hz (amplitude of 3 mm) vibration reduced muscle pain, IL-6 and CK concentrations and knee extension loss caused by DOMS. By contrast, vibrational stimulation at 25 Hz (amplitude of 3 mm) worked poorly. High-frequency vibration training is suggested to be an effective strategy for relieving DOMS after intensive training.

## Data availability statement

The original contributions presented in the study are included in the article/supplementary material, further inquiries can be directed to the corresponding author/s.

## Ethics statement

The studies involving human participants were reviewed and approved by Chengdu Sport University. The patients/participants provided their written informed consent to participate in this study.

## Author contributions

LC and KW: designing this study, writing initial draft and revision, revising language and content, supervision, project administration, and funding acquisition. LC: making figure and table. BH and YY: rechecking the manuscript and putting forward suggestions for amendment. All authors contributed to the article and approved the submitted version.

## Funding

This study was supported by the Sports Medicine Key Laboratory of Sichuan Province, General Administration of Sport of China (2022-A048). The National key research and development plan of china (2019YFF0301704). The project of School of Sports Medicine and Health, Chengdu Sport University (CX21C07).

## Conflict of interest

The authors declare that the research was conducted in the absence of any commercial or financial relationships that could be construed as a potential conflict of interest.

## Publisher's note

All claims expressed in this article are solely those of the authors and do not necessarily represent those of their affiliated organizations, or those of the publisher, the editors and the reviewers. Any product that may be evaluated in this article, or claim that may be made by its manufacturer, is not guaranteed or endorsed by the publisher.
